# An efficient prototype method to identify and correct misspellings in clinical text

**DOI:** 10.1186/s13104-019-4073-y

**Published:** 2019-01-18

**Authors:** T. Elizabeth Workman, Yijun Shao, Guy Divita, Qing Zeng-Treitler

**Affiliations:** 1 The George Washington University, Biomedical Informatics Center, 2600 Virginia Ave, Suite 506, Washington, DC 20037 USA; 2 Division of Epidemiology, University of Utah School of Medicine, 295 Chipeta Way, Salt Lake City, UT 84132 USA

**Keywords:** Spelling analysis, Spelling correction, Clinical text, Word embeddings, Word2Vec

## Abstract

**Objective:**

Misspellings in clinical free text present challenges to natural language processing. With an objective to identify misspellings and their corrections, we developed a prototype spelling analysis method that implements Word2Vec, Levenshtein edit distance constraints, a lexical resource, and corpus term frequencies. We used the prototype method to process two different corpora, surgical pathology reports, and emergency department progress and visit notes, extracted from Veterans Health Administration resources. We evaluated performance by measuring positive predictive value and performing an error analysis of false positive output, using four classifications. We also performed an analysis of spelling errors in each corpus, using common error classifications.

**Results:**

In this small-scale study utilizing a total of 76,786 clinical notes, the prototype method achieved positive predictive values of 0.9057 and 0.8979, respectively, for the surgical pathology reports, and emergency department progress and visit notes, in identifying and correcting misspelled words. False positives varied by corpus. Spelling error types were similar among the two corpora, however, the authors of emergency department progress and visit notes made over four times as many errors. Overall, the results of this study suggest that this method could also perform sufficiently in identifying misspellings in other clinical document types.

**Electronic supplementary material:**

The online version of this article (10.1186/s13104-019-4073-y) contains supplementary material, which is available to authorized users.

## Introduction

Misspellings in clinical text are common, in some instances constituting 5% of all content [1], and over 17% of content addressing a particular domain [2]. Spelling irregularities in clinical text exceed those in other types of text [3] and can significantly affect natural language processing tasks like in part-of-speech tagging [4], drug extraction [5], information retrieval [6], and drug-drug interaction alerts [2]. In studies, Ruch et al. [6] found that even a small amount of misspellings, primarily consisting of common errors, adversely affected information retrieval in clinical text.

Word Embedding (a technique mapping words to real number vectors) facilitated by Word2Vec models [7], holds promise in identifying words with both correct and incorrect spelling forms. Word2Vec models implement neural networks of a single hidden layer to create word vectors, using either a skip-gram or continuous bag of words (CBOW) approach. The skip-gram approach identifies multiple words in a contextual window, given a single word, whereas CBOW identifies a single word given all the other context words in the window. The end-product of either method is the identification of words found in similar contexts.

The Veterans Health Administration (VHA) is the largest integrated health care system in the world, providing care to over 8 million patients each year at over 1243 facilities [8]. Efforts to computerize VHA data began in the 1970s, leading to the creation of VistA, one of the first electronic medical record (EMR) systems [9], and by consequence, the creation of a vast electronic clinical data resource, which VHA maintains in their Corporate Data Warehouse (CDW). These data are made available for research activities through the Veterans Affairs Informatics and Computing Infrastructure (VINCI), a secure platform enabling data research.

We created a prototype method to identify correct and incorrect spelling forms of words in clinical text, using Word2Vec, Levenshtein edit distance [10], the SPECIALIST lexicon [11], and corpus word frequencies, with an eye toward NLP practitioners and their work. We hypothesized that a frequent spelling of a given word would be key in identifying its correctly spelled form, an idea already explored in prior research [12, 13], and that Word2Vec similarity values, edit distance constraints, and a lexical aid could enable identification of its misspellings. Our corpora consisted of randomly-selected surgical pathology notes, and emergency department visit and progress notes from VINCI. We tested our method on these two corpora in order to gauge performance on different types of content. Three annotators assessed output. We measured the positive predictive value, performed an error analysis, and analyzed the output to characterize misspellings according to common error types.

## Main text

### Methods

#### Data procurement and preparation

We extracted two corpora, 50,000 randomly selected surgical pathology notes (SP), and 26,786 emergency department visit and progress notes (EDVP), from the Corporate Data Warehouse, made available through VINCI. To gain relevant information regarding word frequencies, we tokenized each corpus. We removed words appearing in a standard stoplist, which consisted of common functional words (e.g., articles, prepositions). Tokens consisting of only upper case characters, or containing digits, or consisting of less than four characters were also removed, and the remaining were transformed to lower case. These combined actions enabled removal of non-information bearing words. Superfluous punctuation was also removed. Token frequency in each corpus was computed, identifying and storing the 1000 most frequent words in each corpus.

#### Method pipeline

After preliminary testing to determine the most effective hyperparameters, we trained a Word2Vec model for each corpus, implementing the Gensim Word2Vec library in Python, using a dimension of 500 in the hidden layer, and the CBOW algorithm [14], with a maximun window size of 5 words around a given target word. In these models, we also limited processing to words that occurred at least five times in their respective corpus. Each model implemented 10 training epochs. We used the same hyperparameters to train each model.

**Fig. 1:**
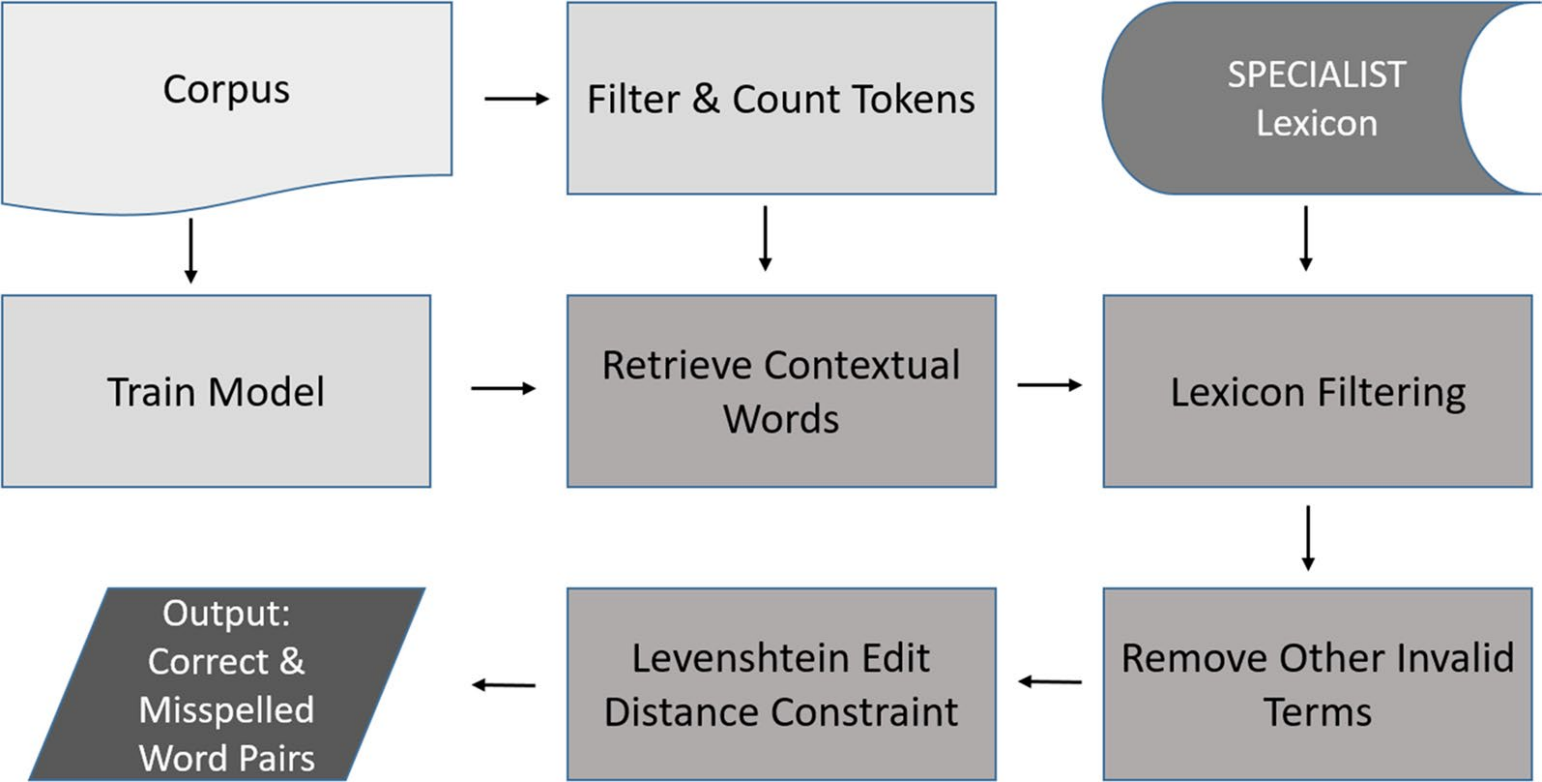
Method pipeline

The 1000 most frequent words in each corpus served as target terms, and for each of these the method retrieved the most similar words (1000 word retrieval maximum), according to Word2Vec’s similarity algorithm. Of these retrieved contextual words, those with standard spellings were removed by comparing them to entries in the SPECIALIST Lexicon, a resource providing standard forms of biomedical terms. The method then removed contextual words that ended with, began with, or contained specific punctuation characters, words that contained digits, and words that were less than four characters in length. Preliminary analysis showed that these smaller words tended to be legitimate abbreviations. Those that remained were transformed to lower case. The method then applied a Levenshtein edit distance of one to three characters of transformation, comparing each candidate contextual word to its matching target word to identify misspellings. Preliminary testing indicated that this edit distance was the most efficient to identify misspellings of the target word at hand, regardless of its length. Figure 1 provides a graphical representation of the method.

#### Output evaluation

Three authors (GD, TEW, QZ) annotated the method’s output for each corpus. For each target word and its potential misspelling, the annotators considered two questions to identify true and false positive output:

- Is the target word a valid, correctly spelled term?
- Is the candidate a misspelling of the target word?

To answer these questions, annotators could use a reference standard, such as a dictionary or lexicon. Candidate terms could not be a valid spelling of another word. Inflections were regarded as different words, i.e., not the same as the given target word. An affirmative answer to both of the questions indicated a true positive finding. We calculated Fleiss’ Kappa [15] to assess inter-annotator agreement. Disagreements were settled by majority rule. If an annotator suspected a candidate term was a true misspelling, but was unsure (by answering “maybe” or “?”) its usage was reviewed in the original corpus.

### Results

#### Raw output for each corpus

The method produced greater output on the most frequent 1000 words from the EDVP corpus, despite its smaller size. In total, there were 235 potential variants identified in the EDVP corpus, as compared to 53 in the SP corpus. There was also a tendency for certain words to have multiple misspelled forms, especially in the EDVP corpus, where approximately 45% of the method’s output consisted of multiple misspelled forms for 38 words. In the SP corpus, approximately 30% of the output consisted of multiple misspelled forms for 7 words. In EDVP, there were 8 different variations of the word “presents”; in SP, there were 4 different variations of the word “received”.

#### Inter-rater agreement

The Fleiss’ Kappa scores were 0.533 for output from the SP corpus and 0.466 for output from the EDVP corpus. The annotators reached moderate agreement in annotating both outputs [16]. The authors felt these scores were sufficient for this exploratory study. Because disagreements were resolved by majority rule, the final classifications of true or false positive for output were the opinions of at least two annotators.

#### Method performance

The positive predictive value (*True Positives*/(*True Positives *+* False Positives*)) for the SP and the EDVP corpora were 0.9057 and 0.8979, respectively. More true misspellings were found in the EDVP corpus than the SP corpus. Additional file 1 includes the true positive and false positive outputs. The following example illustrates an instance of the prototype method successfully identifying a misspelling. “Suicidal”, a correctly spelled term and frequent word in the EDVP corpus, was extracted as a target input word. It was matched to another word in the EDVP corpus, “sucidal”, because they appear in similar contexts in the corpus, manifest by the Word2Vec word embedding similarity value of “sucidal” to “suicidal”. Because it was not in the SPECIALIST Lexicon, and was within the set Levenshtein edit distance, “sucidal” was correctly identified as a misspelling of “suicidal”. In output these were represented as a pair: “suicidal, sucidal”.

#### Error analysis of output (FPs)

All the 29 false positive findings could be classified as different words that were spelled correctly, misspellings of other words besides the given target terms, noise or a nonsensical term, or a colloquial or slang version of the target term that the annotators thought was common enough to be understood as such (Table 1).

**Table 1:** False positives’ types and frequencies

Error type	Surgical pathology notes	Emergency visit and progress notes
Different word, spelled correctly	3 (60%)	9 (37.5%)
Misspelling of different word	0 (0%)	12 (50%)
Alternative form of noisy term	2 (40%)	0 (0%)
Slang equivalent	0 (0%)	3 (12.5%)
Totals	5 (100%)	24 (100%)

#### Characterizing misspellings in the corpora

To categorize spelling errors in each corpus, we separated the true positive output according to common error type: insertion, omission, transposition, wrong letter, or mixed/multiple error types (Table 2). While there were more misspellings identified in the EDVP corpus, misspelling types by percentage were similar across corpora.

**Table 2:** Spelling error types by corpus and frequency

Misspelling type	Surgical pathology notes	Emergency visit and progress notes
Insertion	10 (20.8%)	32 (15.2%)
Omission	25 (52.1%)	103 (48.8%)
Transposition	8 (16.7%)	40 (19%)
Wrong letter	2 (04.1%)	27 (12.8%)
Multiple/mixed	3 (06.3%)	9 (04.2%)
Totals	48 (100%)	211 (100%)

### Discussion

#### Output and method performance

Output volume varied, yet the prototype method achieved comparable performance for each corpus. There was more output for the EDVP corpus than the SP corpus, despite its smaller size. Positive predictive values of 0.9057 (SP) and 0.8979 (EDVP) indicate comparatively good performance on the two different document types. The similar, high positive predictive values suggest that this method may perform well for multiple types of clinical text. More research is needed to determine performance for other document types.

#### Dual task performance

The task of only identifying misspellings is not as difficult as correcting them. This method performs both tasks relatively well. This is, in part, due to its novel application of Word2Vec, which ranks contextual words by similarity values, implementing a preset cutoff, here being a maximum of 1000 words per target word. This is analogous to leveraging information retrieval relevance ranking in identifying similar documents, but applied at the finer-granularity level of words. Combining this with the technique of using the most frequent spelling of the given target word in the corpus as its correct form, lexical filtering, plus applying the Levenshtein edit distance constraint of one to three characters, the prototype method effectively identified matching pairs of correctly spelled and misspelled words. If someone were using the keyword “apetite” in searching the EDVP corpus, this method could both inform the searcher that the likely correct keyword was “appetite”, and that the corpus also included the misspellings “apettite” and “appetitie” for this concept (Additional file 1).

#### Error analysis

The majority of false positive output (3 FPs) for the SP corpus fell into Category 1 (Different word, spelled correctly), but the rest (2 FPs) were in Category 3 (Alternative form of noisy term). The majority of false positive output for the EDVP corpus fell into Category 2 (Misspelling of different word: 12 FPs), but there were also relatively significant amounts in Category 1 (9 FPs) and Category 4 (Slang equivalent: 3 FPs). Terms in Categories 1 and 4 could be added to the SPECIALIST Lexicon. Category 1 terms would definitely be of use. Officials at the National Library of Medicine would need to decide whether slang terms would enrich the SPECIALIST Lexicon.

#### Characterizing misspellings

The most common misspelling type in both corpora was letter omission (Table 2). Letter insertion and transposition were also common in each corpora, with wrong letter and multiple/mixed errors less common. However, there were over four times the total spelling errors in the EDVP corpus. These values suggest that authors of these two corpora tend to make similar mistakes, but those writing emergency visit and progress notes make more mistakes. This may be due to one or more reasons, including different environmental conditions in which the various authors work, and presents an interesting direction for future research. More research involving other document types may provide further insight into the types and frequencies of errors made in clinical text.

### Conclusion

We developed a prototype method to detect and correct misspellings in clinical text. This method uses a pipeline framework to identify correctly and incorrectly spelled word pairs by leveraging term frequencies, Word2Vec, use of the SPECIALIST Lexicon, and a Levenshtein edit distance constraint. An implementation of the method achieved 0.9057 and 0.8979 positive predictive values on two separate corpora. These promising results suggest the need for expanded research regarding this method.

## Limitations

This was only an exploratory study, using small corpora of surgical pathology and emergency department documents. More research is needed to determine the method’s performance for other document types. Because the method uses frequencies, performance for very infrequent terms would likely be affected.

## Additional file

**Additional file 1.** True and false positive findings. This file includes true positive and false positive output of the prototype method. It is organized by corpus and classification type, and lists the given target word and output term, indicated as “Misspelling” or “False Positive”.

## Abbreviations

CBOW: continuous bag of words
CDW: Corporate Data Warehouse
EDVP: Emergency Department Visit and Progress Notes
EMR: electronic medical record
FP: false positive
SP: surgical pathology notes
TP: true positive
VHA: Veterans Health Administration
VINCI: Veterans Affairs Informatics and Computing Infrastructure
