# Supplementary material for: An efficient prototype method to identify and correct misspellings in clinical text
Source: BMC Res Notes. 2019 Jan 18;12:42. doi: 10.1186/s13104-019-4073-y (PMC6339425; doi:10.1186/s13104-019-4073-y)
Supplement: Supplementary file 1 — Additional file 1. True and false positive findings. This file includes true positive and false positive output of the prototype method. It is organized by corpus and classification type, and lists the given target word and output term, indicated as “Misspelling” or “False Positive”. [file 13104_2019_4073_MOESM1_ESM.docx]

| **True Positives: SP** | | | |
| --- | --- | --- | --- |
| **Target Word** | **Misspelling** | **Target Word** | **Misspelling** |
| atypia | atyical | mucosa | muocsa |
| atypical | atyical | notified | notifed |
| cassettes | casettes | o'clock | oclock |
| clinical | clincial | patient | patient' |
| consistent | consistant | patient's | patient' |
| consistent | consisent | patient's | patent's |
| consultation | consultaion | pylori | h.pylori |
| dimension | dimention | qualified | qualifed |
| dimensions | dimention | received | recieved |
| ellipse | elipse | received | receieved |
| entire | entirley | received | b.received |
| entirely | entirley | received | a.received |
| entirety | entirley | requisition | requistion |
| entirety | entirey | reviewed | reveiwed |
| formalin | formlain | reviewed | reviwed |
| gleason | gleason's | significant | signficant |
| greatest | greastest | submitted | submited |
| greatest | greates | surface | suface |
| identification | identfication | surfaces | suface |
| identified | identifed | suspicious | suspicous |
| inflamed | inflammed | tests | test's |
| involved | univolved | thyroid | thryoid |
| irregularly | irregulary | uninvolved | univolved |
| labelled | labled | notified | notifed |

| **True Positives: EDVP** | | | |
| --- | --- | --- | --- |
| **Target Word** | **Misspelling** | **Target Word** | **Misspelling** |
| abdominal | abdomenal | appears | apears |
| about | aout | appetite | apetite |
| abuse | aubse | appetite | apettite |
| access | acces | appetite | appetitie |
| acetaminophen | acetamenphen | appropriately | appropiately |
| acute | actue | approx | aprox |
| admit | admitt | approx | apprx |
| admit | admite | approximately | approximatley |
| admits | admitts | asked | aksed |
| admitted | admited | assessment | assesment |
| admitted | admnitted | assessment | assessement |
| advised | adviced | assigned | asigned |
| advised | adivsed | available | availabe |
| agitation | aggitation | because | becuase |
| agreed | agress | before | befor |
| agrees | agress | behavior | behav |
| albuterol | albuteral | bilaterally | bialterally |
| alcohol | alchol | bilaterally | bilateraly |
| alcohol | alcohal | bowel | bowell |
| alcohol | alchohol | brought | brough |
| appearance | appearence | brought | broght |

| **True Positives: EDVP** | | | |
| --- | --- | --- | --- |
| **Target Word** | **Misspelling** | **Target Word** | **Misspelling** |
| brought | broiught | future | furture |
| chloride | chlorid | given | givne |
| cleared | clared | health | helath |
| complaint | complaitn | hearing | hearling |
| complaint | complant | homicidal | homocidal |
| complaint | complaintof | hydroxyzine | hydroxizine |
| complaints | compliants | hygiene | hygeine |
| cooperative | coperative | impaired | impeared |
| current | curent | improvement | improvment |
| cyclobenzaprine | cyclobenzprine | including | includng |
| cyclobenzaprine | cyclobenzaprene | increase | incrase |
| cyclobenzaprine | cyclobenaprine | increased | incrased |
| decreased | decresed | increasing | increaseing |
| denied | deneis | information | infomration |
| denies | deneis | inhale | inhl |
| depressive | depresive | initial | intial |
| difficulty | difficutly | initially | intially |
| discussed | discused | initially | initally |
| discussed | discsussed | instructed | instucted |
| discussed | discusssed | instructions | intructions |
| drinking | driking | instructions | instuctions |
| earlier | eariler | intent | intnet |
| egfr | egfram | judgment | judjment |
| egfr | egfrafr | likely | likley |
| elevated | elavated | likely | likly |
| erythema | erythmea | management | managment |
| evaluated | evaluted | medically | medicaly |
| evaluation | evaluatin | medications | medicaitons |
| evaluation | evalaution | mellitus | melli |
| evaluation | evaulation | mention | menti |
| evaluation | evalation | mention | mentio |
| exacerbation | exacerbaton | methadone | methodone |
| exacerbation | excerbation | mildly | mildy |
| exacerbation | exaccerbation | movements | movments |
| extremities | extremeties | multiple | mutiple |
| extremities | extremties | multiple | mutliple |
| extremities | extrmities | multiple | muliple |
| extremities | extremeities | multiple | mulitple |
| extremities | extremites | muscle | musle |
| extremity | extrimity | muscle | muslce |
| extremity | extermity | numbness | numnbess |
| follow | follw | offered | offerred |
| from | fron | onset | onsetmay |
| further | futher | ordered | orderd |

| **True Positives: EDVP** | | | |
| --- | --- | --- | --- |
| **Target Word** | **Misspelling** | **Target Word** | **Misspelling** |
| oriented | oreinted | reported | reprts |
| palpation | palpatin | reports | reprots |
| panic | panick | reports | rpeorts |
| persist | perist | requesting | requestes |
| persistent | persistant | requesting | requsting |
| persistent | peristant | review | reviwed |
| persistent | persitent | reviewed | reviwed |
| pleasant | pleasnt | reviewed | reveiwed |
| pleasant | pleasnat | reviewed | reviewd |
| pleasant | pleasatn | rhythm | rythm |
| pleasant | plesant | safety | safty |
| pleasant | pleasent | self | slef |
| podiatry | poditary | separated | seperated |
| polysubstance | polysusbtance | similar | similiar |
| polysubstance | polysubtance | similar | simialr |
| positive | postive | speech | speach |
| practitioner | practitoner | strength | strenth |
| practitioner | practioner | strength | strenght |
| prescribed | prescibed | substance | subtance |
| prescribed | presribed | suicidal | sucidal |
| presentation | presenation | suicide | sucide |
| presented | presentes | swelling | swellling |
| presents | prsents | thoughts | thougts |
| presents | presnets | throat | thoat |
| presents | presnts | towards | twds |
| presents | presentes | trazodone | trazadone |
| presents | presentw | until | untill |
| presents | presens | upper | uppper |
| presents | presetns | urinary | urin |
| presents | presentsto | usual | usuall |
| psychomotor | psychmotor | vascular | vascualar |
| psychomotor | psychomoter | verbalized | vebalized |
| received | recieved | verbalized | verbilizes |
| recommended | recomended | verbalizes | verbilizes |
| recommended | recommeded | vicodin | vicoden |
| recommended | recommened | volume | volumn |
| refill | refil | vomiting | vomitting |
| refills | refil | when | whne |
| refills | refils | withdrawal | withdrawl |
| replacement | replacment | worsening | worsenning |
| report | eport |  |  |

| **False Positives: SP** | | | |
| --- | --- | --- | --- |
| **Target word** | **False Positive** | **Target word** | **False Positive** |
| *+*+*+*+*+*+*+*+* | *+*+*+*+*+*+*+* | wrapped | biowrapped |
| copy | yay | trilineage | trilineal |
| *+*+*+*+*+*+*+* | *+*+*+*+*+*+*+*+* |  |  |

| **False Positives: EDVP** | | | |
| --- | --- | --- | --- |
| **Target word** | **False Positive** | **Target word** | **False Positive** |
| care | tricare | chart | cxry |
| advised | aksed | test | aero |
| want | wanna | labs | lytes |
| verbalizes | vebalized | going | gonna |
| appropriate | appropiately | repeat | reeval |
| presented | presentes | presented | presnts |
| presented | prsents | walk | walkin |
| group | crcp | nicotine | coaine |
| appear | apears | tramadol | trazadone |
| North | epith | persistent | persistance |
| extremities | extremitiy | risperidone | risperdol |
| risperidone | risperidol | serum | aero |
